# Supplementary material for: Neuronal population activity dynamics reveal a low-dimensional signature of operant learning in Aplysia
Source: Commun Biol. 2022 Jan 24;5:90. doi: 10.1038/s42003-022-03044-1 (PMC8786933; doi:10.1038/s42003-022-03044-1)
Supplement: Supplementary file 2 — Reporting Summary [file 42003_2022_3044_MOESM2_ESM.pdf]

nature portfolio

Corresponding author(s): John H. Byrne

Last updated by author(s): Dec 17, 2021

Reporting Summary

Nature Portfolio wishes to improve the reproducibility of the work that we publish. This form provides structure for consistency and transparency in reporting. For further information on Nature Portfolio policies, see our Editorial Policies and the Editorial Policy Checklist.

Statistics

For all statistical analyses, confirm that the following items are present in the figure legend, table legend, main text, or Methods section.

n/a	Confirmed	
☐	☒	The exact sample size (*n*) for each experimental group/condition, given as a discrete number and unit of measurement
☐	☒	A statement on whether measurements were taken from distinct samples or whether the same sample was measured repeatedly
☐	☒	The statistical test(s) used AND whether they are one- or two-sided<br>*Only common tests should be described solely by name; describe more complex techniques in the Methods section.*
☒	☐	A description of all covariates tested
☐	☒	A description of any assumptions or corrections, such as tests of normality and adjustment for multiple comparisons
☐	☒	A full description of the statistical parameters including central tendency (e.g. means) or other basic estimates (e.g. regression coefficient) AND variation (e.g. standard deviation) or associated estimates of uncertainty (e.g. confidence intervals)
☐	☒	For null hypothesis testing, the test statistic (e.g. *F*, *t*, *r*) with confidence intervals, effect sizes, degrees of freedom and *P* value noted<br>*Give P values as exact values whenever suitable.*
☒	☐	For Bayesian analysis, information on the choice of priors and Markov chain Monte Carlo settings
☒	☐	For hierarchical and complex designs, identification of the appropriate level for tests and full reporting of outcomes
☒	☐	Estimates of effect sizes (e.g. Cohen's *d*, Pearson's *r*), indicating how they were calculated

Our web collection on statistics for biologists contains articles on many of the points above.

Software and code

Policy information about availability of computer code

Data collection	Imaging data were acquired using Neuroplex 10.2.2 (RedShirtImaging). Electrophysiological data were acquired simultaneously on Neuroplex 10.2.2 and Clampex 10.4 (Molecular Devices).
Data analysis	Data were analyzed using Sigmaplot 12 (Systat Software) and Matlab R2020b (Mathworks). Non-negative matrix factorization was performed using the seqNMF package (https://github.com/FeeLab/seqNMF; Mackevicius et al.28). Custom algorithms are fully described in the Methods section, and corresponding code is available on Dryad/Zenodo (doi:10.5061/dryad.m37pvmd3q) and Github (https://github.com/Byrne-Lab/costa_et_al_OC_VSD).

For manuscripts utilizing custom algorithms or software that are central to the research but not yet described in published literature, software must be made available to editors and reviewers. We strongly encourage code deposition in a community repository (e.g. GitHub). See the Nature Portfolio guidelines for submitting code & software for further information.

Data

Policy information about availability of data

All manuscripts must include a data availability statement. This statement should provide the following information, where applicable:

- Accession codes, unique identifiers, or web links for publicly available datasets
- A description of any restrictions on data availability
- For clinical datasets or third party data, please ensure that the statement adheres to our policy

Data underlying the study have been deposited to Dryad (doi:10.5061/dryad.m37pvmd3q). The dataset includes activity for all recorded neurons and source data for the plots in this article.

Field-specific reporting

Please select the one below that is the best fit for your research. If you are not sure, read the appropriate sections before making your selection.

[x] Life sciences [ ] Behavioural & social sciences [ ] Ecological, evolutionary & environmental sciences

For a reference copy of the document with all sections, see nature.com/documents/nr-reporting-summary-flat.pdf

Life sciences study design

All studies must disclose on these points even when the disclosure is negative.

Sample size	Sample sizes of N = 7 per group were deemed sufficient based on preliminary results of the operant conditioning training protocol.
Data exclusions	Data were not excluded from analyses. However, preparations were discarded when they did not meet pre-established criteria for training. Criteria are described in the Methods section under "In vitro operant conditioning".
Replication	Experimental procedures and findings were reliably reproduced across multiple animals.
Randomization	Animals from the same batch were paired and randomly assigned to experimental groups.
Blinding	Data analyses were performed while blinded to the training history of each preparation.

Reporting for specific materials, systems and methods

We require information from authors about some types of materials, experimental systems and methods used in many studies. Here, indicate whether each material, system or method listed is relevant to your study. If you are not sure if a list item applies to your research, read the appropriate section before selecting a response.

Materials & experimental systems

n/a	Involved in the study	
[x]	[ ]	Antibodies
[x]	[ ]	Eukaryotic cell lines
[x]	[ ]	Palaeontology and archaeology
[ ]	[x]	Animals and other organisms
[x]	[ ]	Human research participants
[x]	[ ]	Clinical data
[x]	[ ]	Dual use research of concern

Methods

n/a	Involved in the study	
[x]	[ ]	ChIP-seq
[x]	[ ]	Flow cytometry
[x]	[ ]	MRI-based neuroimaging

Animals and other organisms

Policy information about studies involving animals; ARRIVE guidelines recommended for reporting animal research

Laboratory animals	This study used juvenile Aplysia californica (25-50g). Aplysia are hermaphroditic molluscs. Laboratory animals were obtained from National Resource for Aplysia of the University of Miami (Miami, FL).
Wild animals	Wild Aplysia were obtained from South Coast Bio-Marine LLC (San Pedro, CA).
Field-collected samples	N/A
Ethics oversight	Animal care was in accordance with guidelines and overseen by the institutional Animal Welfare Committee. NIH or institutional guidelines for food deprivation protocols do not apply to Aplysia used in the present study because they are marine invertebrates.

Note that full information on the approval of the study protocol must also be provided in the manuscript.
